# Machine learning-based cluster analysis of immune cell subtypes and breast cancer survival

**DOI:** 10.1038/s41598-023-45932-4

**Published:** 2023-11-03

**Authors:** Zhanwei Wang, Dionyssios Katsaros, Junlong Wang, Nicholetta Biglio, Brenda Y. Hernandez, Peiwen Fei, Lingeng Lu, Harvey Risch, Herbert Yu

**Affiliations:** 1 Cancer Epidemiology Program, University of Hawaii Cancer Center, 701 Ilalo Street, Honolulu, HI 96813 USA; 2 Department of Surgical Sciences, Gynecology, AOU Città della Salute, University of Torino, Turin, Italy; 3 Department of Molecular Biosciences and Bioengineering, University of Hawaii at Manoa, Honolulu, HI USA; 4 Division of Obstetrics and Gynecology, Department of Surgical Sciences, University of Torino School of Medicine, Mauriziano Hospital, Turin, Italy; 5 Cancer Biology Program, University of Hawaii Cancer Center, Honolulu, HI USA; 6 Department of Chronic Disease Epidemiology, Yale School of Public Health, New Haven, CT USA

**Keywords:** Cancer, Risk factors

## Abstract

Host immunity involves various immune cells working in concert to achieve balanced immune response. Host immunity interacts with tumorigenic process impacting disease outcome. Clusters of different immune cells may reveal unique host immunity in relation to breast cancer progression. CIBERSORT algorithm was used to estimate relative abundances of 22 immune cell types in 3 datasets, METABRIC, TCGA, and our study. The cell type data in METABRIC were analyzed for cluster using unsupervised hierarchical clustering (UHC). The UHC results were employed to train machine learning models. Kaplan–Meier and Cox regression survival analyses were performed to assess cell clusters in association with relapse-free and overall survival. Differentially expressed genes by clusters were interrogated with IPA for molecular signatures. UHC analysis identified two distinct immune cell clusters, clusters A (83.2%) and B (16.8%). Memory B cells, plasma cells, CD8 positive T cells, resting memory CD4 T cells, activated NK cells, monocytes, M1 macrophages, and resting mast cells were more abundant in clusters A than B, whereas regulatory T cells and M0 and M2 macrophages were more in clusters B than A. Patients in cluster A had favorable survival. Similar survival associations were also observed in other independent studies. IPA analysis showed that pathogen-induced cytokine storm signaling pathway, phagosome formation, and T cell receptor signaling were related to the cell type clusters. Our finding suggests that different immune cell clusters may indicate distinct immune responses to tumor growth, suggesting their potential for disease management.

## Introduction

Host immunity in tumor progression has reemerged as an important focus in cancer research^1^. The new development offers renewed hopes for novel anti-cancer therapies. Recent breakthrough in cancer immunotherapy, especially in the use of immune checkpoint inhibitors (ICI) to treat solid tumors, has invigorated researchers and oncologists in search for new therapeutic modalities to manage recurrent and metastatic malignancies which are otherwise resistant to available treatment^2–4^. However, the success in ICI has not been achieved uniformly for all cancer sites as certain types of cancer do not respond well to the new immunotherapy. ICI has shown promising results in treating melanoma, lung cancer (small cell and non-small cell), renal cell carcinoma, and urothelial carcinoma with significant improvement in clinical outcomes^5–11^, but the efficacy in breast cancer is limited^12,13^. Hormone receptor-positive tumors which are the most common breast cancer do not respond well to immunotherapy; only triple-negative breast cancer (TNBC) appears to have limited responses^14^. Thus, to better understand host immunity in breast cancer, we need to know not only the involvement of different immune and tumor cells, but also their interactions and responses to treatment.

Tumor microenvironment (TME) has been recognized to have significant impacts on cancer cell functions and activities and therefore affect tumor progression and metastasis. In addition to tumor cells and stromal components in TME, many local and infiltrating immune cells also play a crucial role in determining tumor growth and disease outcome^15–17^. Analyzing their configurations and abundances in TME has emerged as important parameters in assessing tumor specimens, predicting disease outcomes, and developing treatment strategies. Studies have shown that infiltrating cytotoxic lymphocytes in TME are associated with the efficacy of immunotherapy^17,18^. TNBC patients with high tumor infiltrating lymphocytes (TIL) are more responsive to ICI, whereas those with hormone receptor-positive breast tumors and low TIL are less responsive^19^. This discrepancy in TIL is explained in part by the differences in somatic mutations which not only reprogram cell signal pathways and metabolisms, but also generate tumor-associated and tumor-specific antigens (TAA, TSA)^20,21^. These altered or mutant molecules induce host immune response by attracting immune cell infiltration and congregation. Characterizing the abundance and composition of immune cell subtypes in tumor samples has shown values in disease prognosis and prediction of treatment responses^22,23^.

Cell sorting by flow cytometry and tissue staining with immunohistochemistry have been used to assess TIL, but these methods have some limitations with respect to tissue accessibility, processing challenges, and subjective evaluation^24^. Recently, computational approaches have been developed for in silico prediction of immune cell subtype abundances based on the readily available gene expression data on tissue transcriptomes. To assess if immune cell subtype clusters are useful for breast cancer prognosis, we analyzed transcriptomic data from several breast cancer datasets using the computation algorithm CYBERSORT^25^. The results of our analyses are presented in this report.

## Results

### Clusters of immune cell subtypes

**Figure 1:**
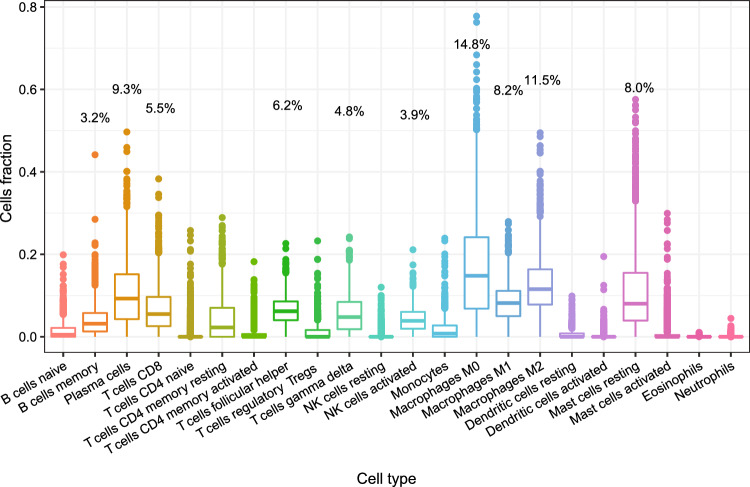
Distributions of immune cell subtypes in METABRIC.

Figure 1 shows the relative abundances of each immune cell subtypes in METABRIC. Over half of the cell subtypes had very low abundances. Cell subtypes with relatively high abundances were M0 macrophages (14.8%), M2 macrophages (11.5%), plasma cells (9.3%), M1 macrophages (8.2%), resting mast cells (8.0%), follicular helper T cells (6.2%), CD8 positive T cells (5.5%), gamma delta T cells (4.8%), activated NK cells (3.9%), and memory B cells (3.2%).

UHC analysis indicated two clusters of immune cell subtypes in METABRIC (Supplementary Fig. 1). One cluster (hcluster 1 or cluster A) was observed in 1113 patients (83.2%), and another (hcluster 2 or cluster B) was in 224 patients (16.8%). Differences in cell subtypes between the two clusters and their comparisons with normal breast tissues are shown in Table 1. Cell subtypes which were significantly different between the two clusters included memory B cells, plasma cells, CD8 positive T cells, resting memory CD4 T cells, activated NK cells, monocytes, M1 macrophages, and resting mast cells, which showed higher abundances in cluster A than cluster B. Cell subtypes with relative abundances higher in cluster B than cluster A were regulatory T cells and M0 and M2 macrophages.

**Table 1:** Two clusters of immune cell subtypes in METABRIC and immune cell subtypes in GTEx breast.

Immune cell subtype	Median % in Cluster A (n = 1113)	Median % in Cluster B (n = 224)	Median % in GTEx (n = 269)	*P* value* Cluster A versus Cluster B	*P* value* Cluster A versus GTEx	*P* value* Cluster B versus GTEx
B cells naïve	0.50	0.49	8.10	0.9907	**4.4E−85**	**1.0E−48**
B cells memory	3.44	2.08	0.00	**5.8E−10**	**6.4E−99**	**3.9E−51**
Plasma cells	10.10	4.86	6.49	**4.1E−22**	**6.1E−07**	0.0084
T cells CD8	6.33	2.71	7.41	**4.7E−29**	0.0092	**2.7E−26**
T cells CD4 naïve	0.00	0.00	0.00	0.0922	**3.1E−13**	**1.7E−07**
T cells CD4 memory resting	2.80	0.00	9.47	**3.3E−08**	**1.2E−42**	**2.5E−45**
T cells CD4 memory activated	0.00	0.00	0.00	**0.0002**	**7.2E−26**	**1.8E−12**
T cells follicular helper	6.22	6.13	2.68	0.1943	**1.3E−26**	**3.0E−13**
T cells regulatory Tregs	0.00	1.28	0.00	**3.4E−15**	**2.8E−05**	**2.1E−22**
T cells gamma delta	4.86	4.25	0.00	0.1071	**4.0E−109**	**4.6E−82**
NK cells resting	0.00	0.00	1.92	**5.1E−13**	**7.4E−114**	**5.7E−26**
NK cells activated	4.32	1.70	2.09	**5.4E−33**	**9.9E−25**	0.011
Monocytes	1.03	0.00	3.16	**5.5E−13**	**2.8E−32**	**2.4E−40**
Macrophages M0	12.40	36.00	0.00	**8.5E−107**	**8.7E−66**	**2.0E−76**
Macrophages M1	8.52	6.46	1.65	**3.1E−09**	**1.6E−86**	**3.9E−33**
Macrophages M2	10.90	15.40	23.79	**1.5E−14**	**7.2E−65**	**3.5E−26**
Dendritic cells resting	0.00	0.00	0.00	**1.1E−15**	**3.4E−22**	0.29
Dendritic cells activated	0.00	0.00	0.00	0.8621	**5.8E−07**	**0.0019**
Mast cells resting	8.98	3.93	11.72	**2.3E−27**	**0.0001**	**5.6E−31**
Mast cells activated	0.00	0.00	0.00	**2.6E−12**	0.0074	**5.2E−14**
Eosinophils	0.00	0.00	0.00	0.0364	**6.3E−15**	0.0023
Neutrophils	0.00	0.00	0.00	0.0605	**2.5E−21**	**2.7E−06**

*Mann–Whitney nonparametric test; bold *p* values < 0.002273 (0.05/22).

Immune cell subtype abundances were very different between normal breasts and breast tumors (Table 1). Compared to normal breasts, less abundant cell types in breast tumors included naïve B cells, resting CD4 memory T cells, resting NK cells, M2 macrophages, and resting mast cells; more abundant cell types in tumor samples were memory B cells, follicular helper T cells, gamma delta T cells, and M0 and M1 macrophages. Different abundances between clusters A and B tumor samples in comparison to normal breasts were plasma cells (higher in A, but lower in B), CD8 T cells (no difference in A, but lower in B), and activated NK cells (higher in A, but no difference in B).

**Figure 2:**
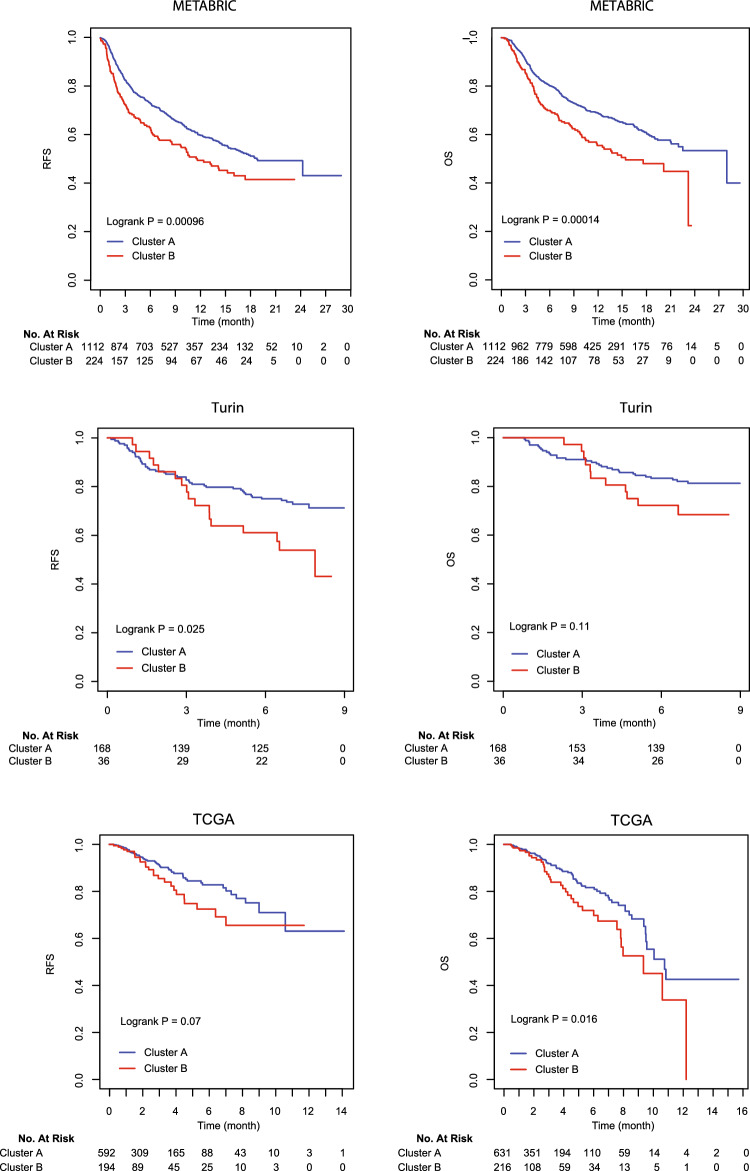
Kaplan–Meier curves on relapse-free survival (RFS) and overall survival (OS) in METABRIC, Turin, and TCGA.

Associations of immune cell clusters with clinical and pathological variables of breast cancer in METABRIC are shown in Table 2. Patients with ER negative tumors or invasive ductal carcinoma were more prevalent in cluster B than in cluster A, and patients in cluster B were also more likely to develop recurrent disease or die. As expected, patients in cluster A had higher immune cytolytic activity or CYT scores compared to those in cluster B. Disease stage, tumor grade, age at diagnosis, PR status, and ERBB2 (HER2) overexpression were not significantly different between the two cell clusters. The cell cluster variable was significantly associated with relapse-free and overall survival (Fig. 2, METABRIC). These associations remained statistically significant in Cox proportional hazards regression models after clinical and pathological variables were adjusted in the analysis, including age at diagnosis, disease stage, tumor grade, tumor histology, and hormone receptor status (Table 3).

**Table 2:** Associations between clinicopathological variables in METABRIC and immune cell clusters.

Clinicopathological variable	Immune cell subtype clusters			*p* value*
	Cluster A, n = 1113 (83.2%)	Cluster B, n = 224 (16.8%)	Total n = 1337	
Mean age (SD)	59.4 (13.1)	59.8 (13.2)	59.4 (13.1)	0.67
Age group				0.91
< 60 years	556 (50.0)	111 (49.6)	667 (49.9)	
≥ 60 years	557 (50.0)	113 (50.4)	670 (50.1)	
Stage				0.41
0	2 (0.2)	1 (0.6)	3 (0.3)	
1	2781 (33.5)	56 (32.2)	334 (33.2)	
2	464 (55.8)	105 (60.3)	569 (56.6)	
3	80 (9.6)	12 (6.9)	92 (9.2)	
4	7 (0.8)	7 (0.7)	0	
Grade				0.11
1	89 (8.3)	13 (6.0)	102 (7.9)	
2	405 (37.7)	70 (32.4)	475 (36.8)	
3	580 (54.0)	133 (61.6)	713 (55.3)	
Histology				**0.006**
Ductal	830 (75.1)	191 (86.0)	1021 (76.9)	
Lobular	96 (8.7)	10 (4.5)	106 (8.0)	
Mixed	128 (11.6)	16 (7.2)	144 (10.9)	
Others	51 (4.6)	5 (2.3)	56 (4.2)	
ER				**0.006**
Positive	799 (71.8)	140 (62.5)	939 (70.2)	
Negative	314 (28.2)	84 (37.5)	398 (29.8)	
PR				0.16
Positive	529 (47.5)	95 (42.4)	624 (46.7)	
Negative	584 (52.5)	129 (57.6)	713 (53.3)	
HER2				0.24
Positive	168(15.1)	27(12.1)	195(14.6)	
Negative	945(84.9)	197(87.9)	1,142(85.6)	
Relapse				**0.003**
No	677(60.9)	112(50.0)	789(59.1)	
Yes	435(39.1)	112(50.0)	547(40.9)	
Death				**0.001**
No	522(46.9)	78(34.8)	600(44.9)	
Yes	591(53.1)	146(65.2)	737(55.1)	
Cytolytic activity				< **0.0001**
CYT score	6.9 (0.7)	6.3 (0.6)	6.8 (0.8)	

*Student’s T-test, Pearson’s Chi-squared test, or Fisher’s exact test where appropriate.

Significant values are in bold.

**Table 3:** Associations between immune cell subtype cluster and breast cancer survival.

Dataset	Relapse-free survival			Overall survival			Relapse-free survival*			Overall survival*		
	HR	95%CI	*P*	HR	95%CI	*P*	HR	95%CI	*P*	HR	95%CI	*P*
METABRIC			**0.001**			**< 0.001**			**0.009**			**0.011**
Cluster A	1			1			1			1		
Cluster B	1.42	1.15–1.74		1.60	1.28–2.00		1.38	1.08–1.76		1.37	1.08–1.75	
Turin study			**0.028**			0.114			0.154			0.358
Cluster A	1			1			1			1		
Cluster B	1.87	1.07–3.27		1.74	0.85–3.47		1.53	0.85–2.73		1.40	0.68–2.89	
TCGA#			0.072			**0.017**			**0.002**			**0.004**
Cluster A	1			1			1			1		
Cluster B	1.54	0.96–2.47		1.63	1.09–2.43		2.24	1.34–3.74		1.93	1.24–3.01	

*Adjusted for age, stage, grade, ER, PR, and histology.

^#^Tumor grade not included in multivariate analysis.

Significant values are in bold.

### Immune cell cluster modeling

We used random forest (RF) to build a prediction model for cell subtype clusters. The RF model was trained with the UHC results in 60% of the METABRIC data, and the model fit well to the UHC clusters with 100% and 98% AUC in the training and testing sets, respectively (Supplementary Fig. 2). Although DNN, elastic net, and stepAIC models were also matched well to UHC, the AUC of RF in the training set was higher than that in other three models. Thus, we used the RF model to predict immune cell clusters in the Turin study and TCGA. The RF predicted cell clusters were analyzed for its associations with patient survival. Similar associations with relapse-free and overall survival were found in the Turin study (Fig. 2), i.e., cluster B associated with poor survival, although the associations were not statistically significant after adjusting for clinicopathological variables (Table 3). Associations between patient survival and immune cell clusters were also observed in TCGA. Patients with immune cell subtypes in cluster B had higher risks for disease recurrence and death compared to those with cell subtypes in cluster A (Fig. 2). The survival associations in TCGA were statistically significant after adjusting for clinicopathological variables (Table 3). No associations between immune cell clusters and ER status or histological types were observed in these validation studies (data not shown).

The importance of each cell type in the RF model was evaluated with mean decreases in accuracy and the Gini coefficient. The top 5 important cell types were M0 and M2 macrophages, CD8 positive T cells, activated NK cells, and resting mast cells (Supplementary Fig. 3). The stepAIC analysis showed a 19-cell model, and the elastic net suggested a 13-cell regression (Supplementary Table 1). Twelve cell types were common in both models, including naïve B cells, memory B cells, plasma cells, CD8 positive T cells, resting memory CD4 T cells, activated memory CD4 T cells, regulatory T cells, activated NK cells, M2 macrophages, resting mast cells, activated mast cells, and neutrophils.

### IPA analysis on DEGs

**Figure 3:**
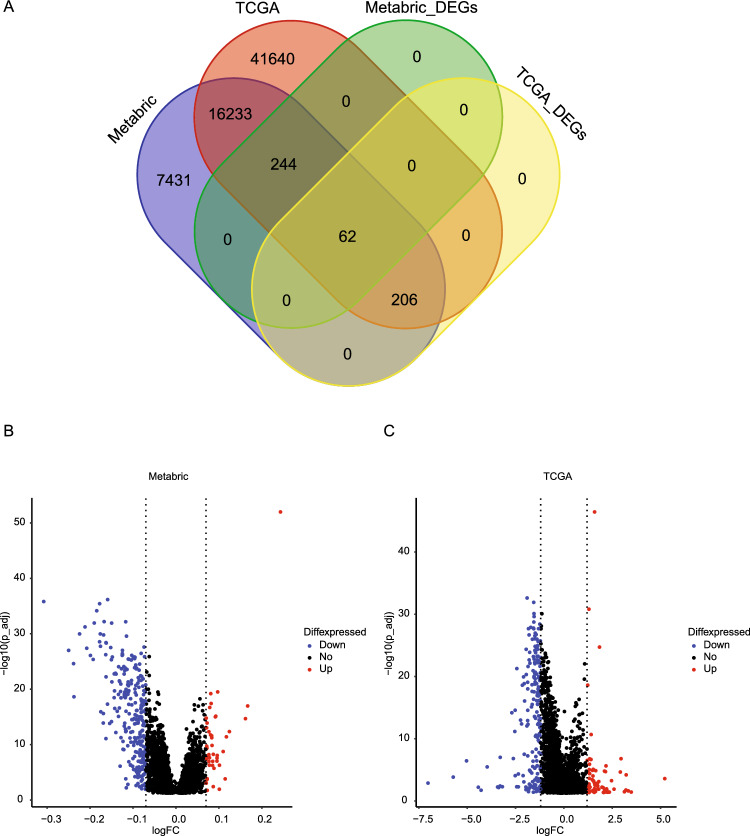
(**A**) Venn diagram of total available genes and DEGs in METABRIC and TCGA; (**B**) Volcano plot of DEGs identified in METABRIC; (**C**) Volcano plot of DEGs identified in TCGA. FC: fold change.

**Figure 4:**
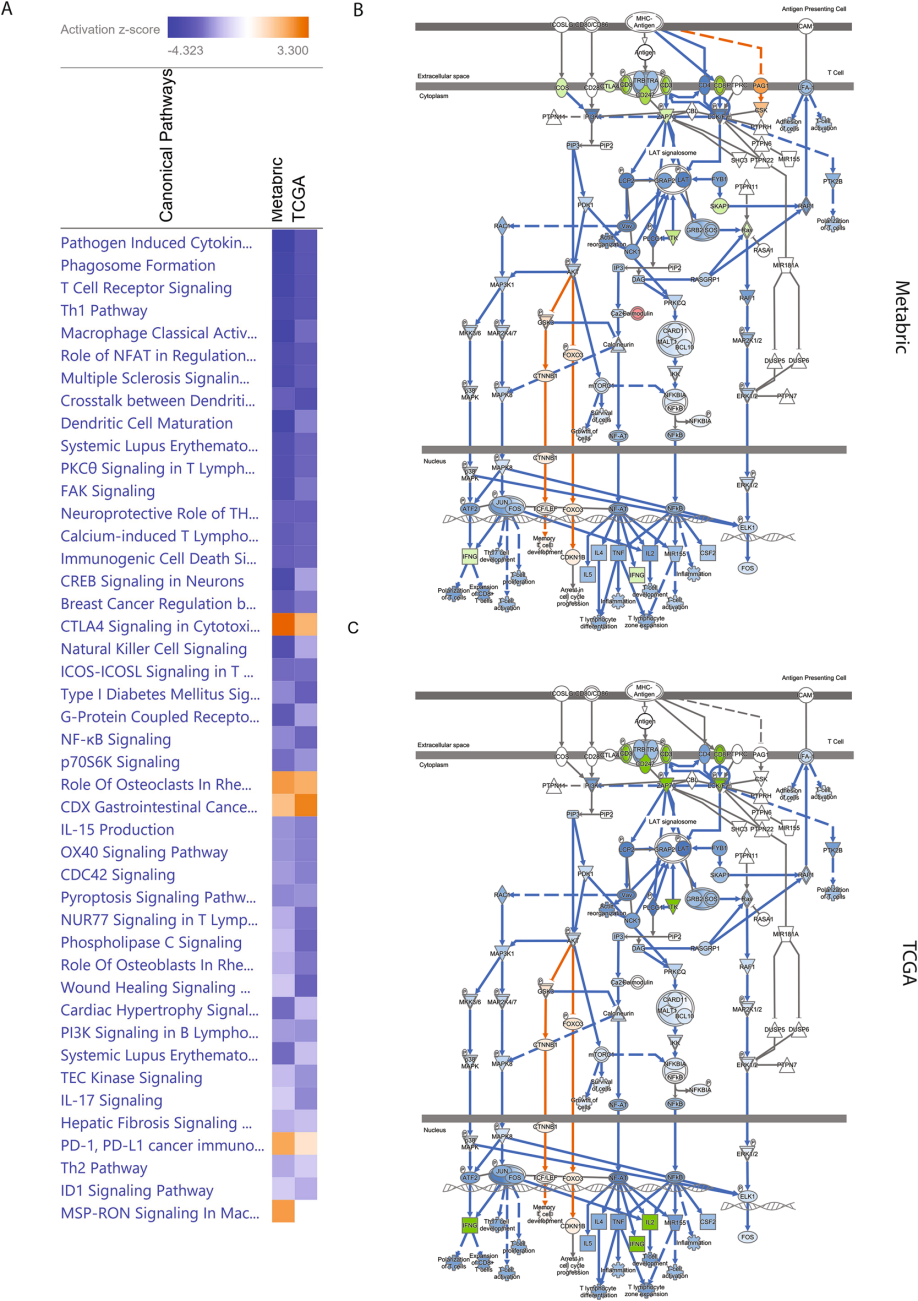
(**A**) Comparison of IPA analysis between METABRIC and TCGA using the ingenuity pathway analysis (IPA, www.qiagen.com/ingenuity); (**B**) T cell receptor signaling predicted IPA in METABRIC; (**C**) T cell receptor signaling predicted by IPA in TCGA.

There were 16,621 genes overlapping between the transcriptomic data of METABRIC and TCGA. IPA was performed on the 268 DEGs in TCGA (absolute log2 fold change at 1.2 or larger for cluster B versus cluster A; BH adjusted *P* < 0.05) (Fig. 3A). Since the expression data in METABRIC had a smaller range and the median fold change was only 1.001 (IQR: 0.996–1.184), we used the absolute log2 fold change at 0.07 as a threshold and selected 306 DEGs for IPA analysis. Volcano plot showed the selected DEGs in METABRIC and TCGA (Fig. 3B,C). Graphical summary of IPA analysis on cell cluster associated DEGs showed that the transcription profiles were similar between METABRIC and TCGA, with most of the signal pathways being downregulated (Supplementary Fig. 4). The top 5 common signal pathways predicted by IPA in METABRIC and TCGA were pathogen induced cytokine storm signaling pathway, phagosome formation, T cell receptor signaling, T helper 1 pathway, and macrophage classical activity, all of which were downregulated (Fig. 4A). The T cell receptor signaling showed the similar patterns of network in METABRIC and TCGA (Fig. 4B,C).

## Discussion

We used CIBERSORT to estimate the relative abundances of 22 immune cell subtypes in breast cancer and normal breast tissues and found significant differences in cell types between tumor and normal tissues. The deconvolution results on cell subtypes were further analyzed in breast cancer (METABRIC) with unsupervised hierarchical clustering, and the analysis suggested two distinct clusters of immune cell subtypes associated with different survival outcomes of breast cancer. These survival associations were replicated independently in our study (Turin) and TCGA when using a random forest model which was trained with the UHC classifications in METABRIC. The survival associations with immune cell clusters appeared to be independent from most known clinical and pathological variables of breast cancer, suggesting the importance of host immunity in determining tumor progression and host-tumor interaction. The machine learning-based cell cluster analyses split the tumor samples into large (83%) and small (17%) groups, which appears to match with the general trend of breast cancer outcome where most patients have a favorable prognosis (> 80%).

Previously, Ali et al. performed hierarchical clustering analysis on immune cell subtypes in 10,988 tumor samples from 56 studies^26^. Their analysis showed 7 clusters in 6071 samples. The authors concluded that there were substantial variations in immune cell subtypes in TME and that tumor characteristics might determine the cell type variability. A recent study by Tekpli et al.^27^ reported 3 clusters of immune infiltration based on the expression of 509 genes, and the clusters were correlated with lymphoid and myeloid infiltration from low to high, with high and low infiltration clusters associated with favorable survival compared to intermediate infiltration. Since the study used a different method to determine tumor immunity, we cannot directly compare the clustering results between the two studies, but both studies indicate that breast cancer may be classified into immunity-based subtypes which have clinical implications in predicting disease prognosis and treatment response.

Going through the cell types in each cluster, we found that memory B cells, plasma cells, CD8 positive T cells, resting memory CD4 T cells, activated NK cells, monocytes, M1 macrophages, and resting mast cells were significantly higher in the favorable cluster (cluster A), whereas regulatory T cells and M0 and M2 macrophages were substantially higher in unfavorable cluster (cluster B). These differentiating cell types appear to be consistent with the current understanding that hot or immune-inflamed TME, which has favorable prognosis and is responsive to immunotherapy, is infiltrated with cytotoxic T cells (CD8 positive T cells), NK cells, and M1 macrophages, whereas cold TME is filled with immunosuppressive lymphocytes like regulatory T cells and tumor-associated macrophages (TAM), M0 and M2^28^. NK cells and CD8 positive T cells are known to be able to suppress tumor growth through their cytotoxic activities^28,29^. Furthermore, CD4 memory T cells and M1 microphages facilitate the effects of NK cells and cytotoxic T cells^30^. Conversely, M2 macrophages and regulatory T cells inhibit the activities of CD4 memory and CD8 cytotoxic T cells, respectively^31^.

We analyzed the cell type data by focusing on immune cells in clusters instead of individual cells because host immunity is complex and involves different mechanisms and diverse cell lineages which give rise to innate versus adaptive, local versus systemic, and cellular versus humoral immunities. These distinct immune activities are carried out by a variety of cell types which work in concert to mount an appropriate immune response^32^. Thus, analyzing any single cell or a few cell types may not reveal enough insights into the interplay between tumor immunogenicity and host immune response as well as the potential impact of their interaction on tumor growth and disease outcome^33^. Ali et al.^26^ assessed individual immune cell subtypes in relation to breast cancer survival by ER status, and the large study found that only two cell types showed consistent associations with survival outcomes, regulatory T cells and M2 macrophages, both of which were associated with poor survival. Although multiple cell type clusters were found in that study, the survival associations with some cell types were generally consistent with those observed in our cluster analysis. For example, Ali et al.^26^ found favorable survival associations with monocytes and memory B cells in ER positive tumors and with CD8 positive T cells in ER negative tumors, as well as unfavorable survival associations with M0 macrophages for ER positive tumors.

TCR stimulation is a fundamental step in most T cell responses. TCR signaling is important for many aspects of T cell regulation, including development, differentiation, activation, proliferation, and survival. Dysregulation of TCR signaling can result in allergy and autoimmune diseases^34^. The molecular mechanism of TCR suppression underlying the link between immune cells in cluster B and breast cancer progression remains to be elucidated.

One limitation of our study is that we cannot assess the temporal and spatial variations of immune cell subtypes in tumor specimens, which is known to play an important role in determining the effect of host immunity and host-immune interplay in addition to cell types^35,36^. Anti-cancer therapies are known to have significant impacts on TME and immune cell infiltration^37^. Our analysis of immune cell subtypes in cluster only reflects the cell composition at the time of initial mastectomy which may be considered as a baseline status of TME that is different from those of post-surgery and during systemic anti-cancer treatment. The other limitation is that our deconvolution was not based on the entire 547 reference genes in LM22. Although not all signature matrix genes are required for deconvolution, the algorithm’s performance is improved with the presence of more signature genes^38^.

## Conclusions

This study applied different machine learning methods to analyze immune cell subtypes in clusters and found two distinct clusters in breast cancer associated with survival outcomes. The survival associations were replicated independently in two additional datasets. Immune cell subtypes which were more abundant in the cluster of favorable prognosis included memory B cells, plasma cells, CD8 positive T cells, resting memory CD4 T cells, activated NK cells, monocytes, M1 macrophages, and resting mast cells, and those less abundant were regulatory T cells, and M0 and M2 macrophages. The immune cell clusters associated with breast cancer progression may involve suppression of pathogen induced cytokine storm signaling pathway, phagosome formation, T cell receptor signaling, T helper 1 cell pathway, and macrophage classical activity pathways. Our finding suggests that immune cell clusters in primary breast cancer may be an important parameter to consider, in addition to individual cell types, when predicting disease outcome and planning treatment strategy.

## Methods

### Study design and participants

Two online datasets on transcriptome, METABRIC and TCGA^39,40^, were used for analysis together with their clinical and follow-up information. METABRIC, downloaded from cBioPortal (https://www.cbioportal.org/)^41,42^, has 1903 breast tumor samples with gene expression data on 24,368 genes measured by a microarray chip from Illumine (Illumina HT-12 v3). The log2 intensity values were used for cell type deconvolution. Clinical data and survival information available for analysis in METABRIC include age at diagnosis, disease stage, tumor grade, histological type, estrogen receptor (ER) status, progesterone receptor (PR) status, ERBB2 (HER2) overexpression, disease recurrence, death, and follow-up time. TCGA RNA-seq data, expressed as fragments per kilobase of exon per million mapped fragments (FPKM), on 1075 breast tumor samples were downloaded from the Genomic Data Commons (GDC) data portal (https://portal.gdc.cancer.gov/)^43^. The corresponding clinical information was downloaded from cBioPortal.

An independent dataset of tumor transcriptomes from 204 breast cancer patients was available from a previous study (Turin) of ours described in detail elsewhere^44^. In brief, we recruited 348 patients who were diagnosed with primary breast cancer and underwent mastectomies in the University Hospital at University of Turin in Italy^45^. Fresh tumor samples were collected during surgery and snap-frozen in liquid nitrogen immediately after resection. Total RNA was extracted, of which 205 were selected for microarray analysis using the Illumina Expression BeadChip (HumanRef-8 v1). The raw expression data (~ .idat) generated by the Illumina microarray assay were processed using GenomeStudio V2011.1. Data was normalized using the function neqc() in R package limma, This function performs normexp background correction using negative controls, then quantile normalizes and finally log2 transforms. The normalized data was ready for CIBERSORT deconvolution of 22 immune cell types^24,46^. Transcriptomic data on normal breast tissues were downloaded from the GTEx Portal (https://www.gtexportal.org/home/datasets) which contains the transcripts per million (TPM) of RNA-seq data on 459 tissue specimens (GTEx Analysis V8).

CIBERSORT estimates the relative abundances of immune cell subtypes in tissue samples. The computation algorithm deconvolutes 22 immune cell subtypes from tumor transcriptomes using reference LM22. LM22 includes the expression of 547 reference genes, of which 475 were available in METABRIC, 444 in the Turin study, 537 in TCGA, and 527 in the GTEx data. CIBERSORT interrogates tumor transcriptome for immune cell subtypes based on the assumption that tissue samples contain mixed cell populations^38^. To evaluate the validity of cell type deconvolution in METABRIC and TCGA, we selected 100 permutations as recommended to achieve statistical rigor without applying quantile normalization. Tumor samples with deconvolution results not significantly different from the null hypothesis (*p* > 0.05) were excluded from final analysis. The null hypothesis assumes no immune cell subtypes present in a tumor sample based on LM22. After removing the samples without significance, we obtained 1337 samples from METABRIC, 848 samples from TCGA, and 269 samples from GTEx qualified for cell type analysis.

### Model development and statistical analysis

We performed unsupervised hierarchical clustering (UHC) analysis on the immune cell subtypes from METABRIC using the ‘hclust’ function with ‘complete’ selection in R. Based on the UHC results, we created a dichotomous variable on cell subtype clusters. Differences in immune cell subtypes between clusters were compared using the Mann–Whitney nonparametric U test. Associations of cell subtype clusters with clinical and pathological variables were analyzed with the Chi-square test. Kaplan–Meier survival curves and log-rank test were used to evaluate survival differences between patients in different immune cell clusters. Cox proportional hazards regression analysis was performed to determine survival associations with immune cell clusters while adjusting for clinicopathological variables. Two-side *p* values < 0.05 were considered statistical significance. All the analyses were performed using R (version 4.0.5).

To predict cell subtype clusters, we tested 4 machine learning models, including random forest (RF), deep neural network (DNN), stepAIC, and elastic net. The models were initially trained based on the UHC results in 60% of METABRIC and then tested in the remaining 40% of the data. METABRIC data were randomly split into training and testing sets. The RF model was developed using the ‘randomForest’ package in R with 500-tree selection. The importance of immune cell subtypes in the model was evaluated by mean decrease in accuracy and the Gini coefficient. The DNN model was trained using the CPU implementation of TensorFlow (version 1.14.0) for 2000 steps with a 7 × 7 hidden layer in Python (version 3.6.13). Regression models of stepAIC and elastic net were developed using the “MASS” and “glmnet” packages in R (version 4.0.3)^47^. Model comparison was made between UHC and each of the 4 machine learning methods using the “pROC” package in R which calculates the receiver operating characteristic (ROC) curves and area under the curve (AUC)^48^. DeLong’s test was used for AUC comparison between models. We also evaluated immune cytolytic activity by calculating the CYT score^49^.

Wilcoxon test was performed for the differentially expressed genes (DEG) analysis between cluster A and cluster B (cluster B vs. cluster A) in METABRIC and TCGA. *P* values were adjusted for the Benjamin-Hochberg correction (BH). The ingenuity pathway analysis (IPA) (www.qiagen.com/ingenuity) was performed on the significant DEGs to explore the signal pathways enriched in cell clusters.

### Supplementary Information

Supplementary Information.

## Data Availability

The TCGA, METABRIC, and Transcriptomic data on normal breast tissues are available in the following website: https://portal.gdc.cancer.gov; https://www.cbioportal.org; https://www.gtexportal.org/home/datasets, respectively. All additional information including Turin data required to reproduce our results is available from the corresponding author upon request.
