# Supplementary material for: Machine learning-based cluster analysis of immune cell subtypes and breast cancer survival
Source: Sci Rep. 2023 Nov 3;13:18962. doi: 10.1038/s41598-023-45932-4 (PMC10624674; doi:10.1038/s41598-023-45932-4)
Supplement: Supplementary file 1 — Supplementary Information. [file 41598_2023_45932_MOESM1_ESM.pdf]

Machine learning-based cluster analysis of immune cell subtypes and breast cancer survival

Zhanwei Wang[1], Dionyssios Katsaros[2], Junlong Wang[1, 3], Nicholetta Biglio[4], Brenda Y. Hernandez[1], Peiwen Fei[5], Lingeng Lu[6], Harvey Risch[6], Herbert Yu[1, *]

1. Cancer Epidemiology Program, University of Hawaii Cancer Center, Honolulu, Hawaii
2. Department of Surgical Sciences, Gynecology, AOU Città della Salute, University of Torino, Turin, Italy
3. Department of Molecular Biosciences & Bioengineering, University of Hawaii at Manoa, Honolulu, Hawaii
4. Department of Surgical Sciences, Division of Obstetrics and Gynecology, University of Torino School of Medicine, Mauriziano Hospital, Turin, Italy
5. Cancer Biology Program, University of Hawaii Cancer Center, Honolulu, Hawaii
6. Department of Chronic Disease Epidemiology, Yale School of Public Health, New Haven, Connecticut

Corresponding author:
Herbert Yu, MD, PhD
University of Hawaii Cancer Center
701 Ilalo Street, Honolulu, HI 96813
Email: hyu@cc.hawaii.edu

Supplementary Figure 1. Heatmap to show the results of unsupervised hierarchical clustering analysis using heatmap.2 function from gplots package in R (version 4.0.3). Y-axis: immune cell subtypes. X-axis: tumor samples; red – high abundance of cell type; green – low abundance of cell type; Bottom bar (hcluster): cell cluster; red – Cluster B; green – Cluster A.

Supplementary Figure 2. Receiver operating characteristics (ROC) curves and areas under the curve (AUC) when comparing cell cluster results of unsupervised hierarchical clustering (UHC) with random forest (RF), deep neural network (DNN), elastic net, and stepAIC in the METABRIC training and METABRIC testing sets.

Supplementary Figure 3. Assessment of cell type importance in random forest analysis by mean decreases in accuracy and the Gini coefficient.

Supplementary Figure 4. A. The ingenuity pathway analysis (IPA, www.qiagen.com/ingenuity) graphical summary of DEGs from METABRIC; B. IPA graphical summary of DEGs from TCGA.

Supplementary Figure 1.

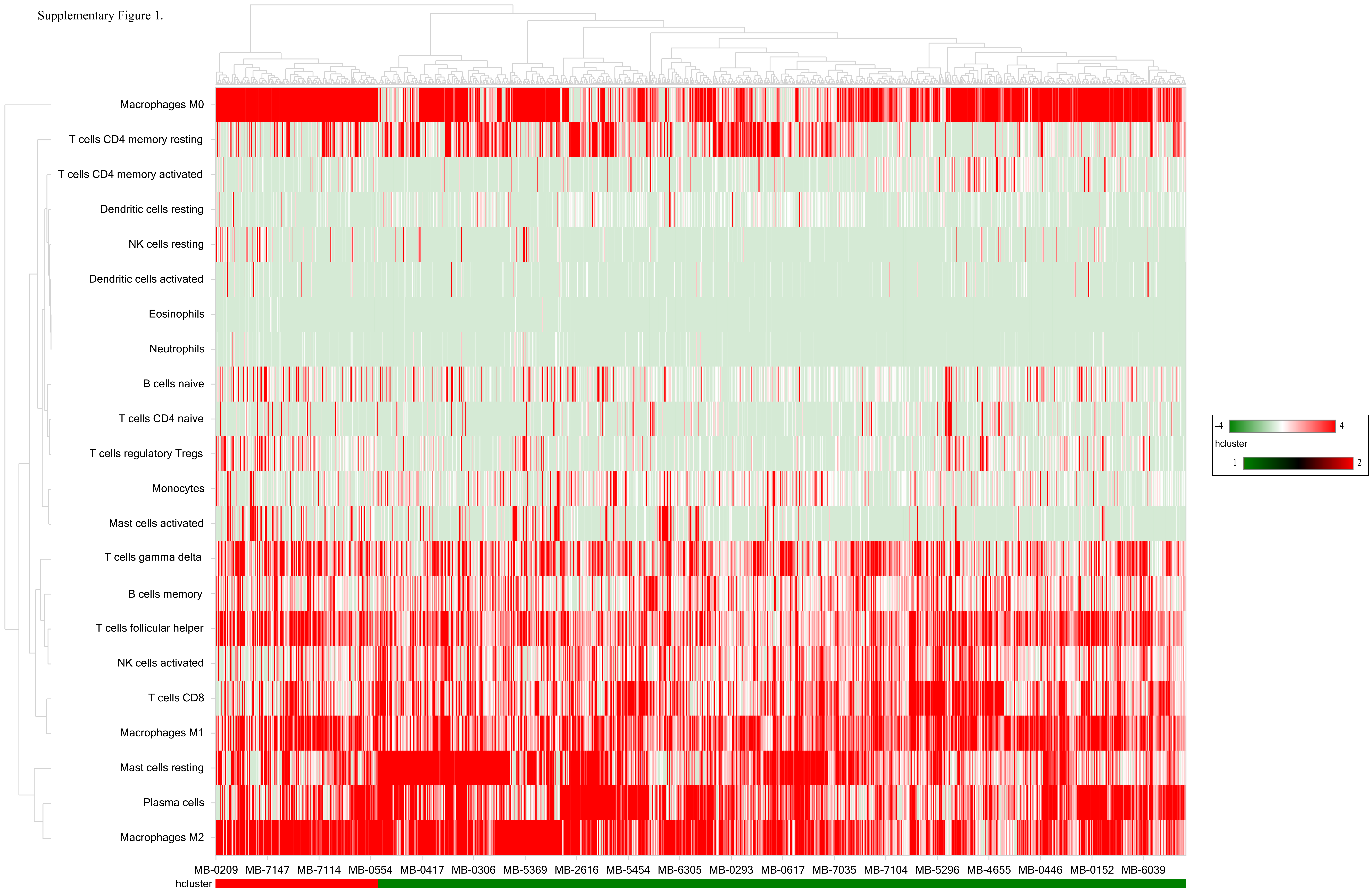

Supplementary Figure 2

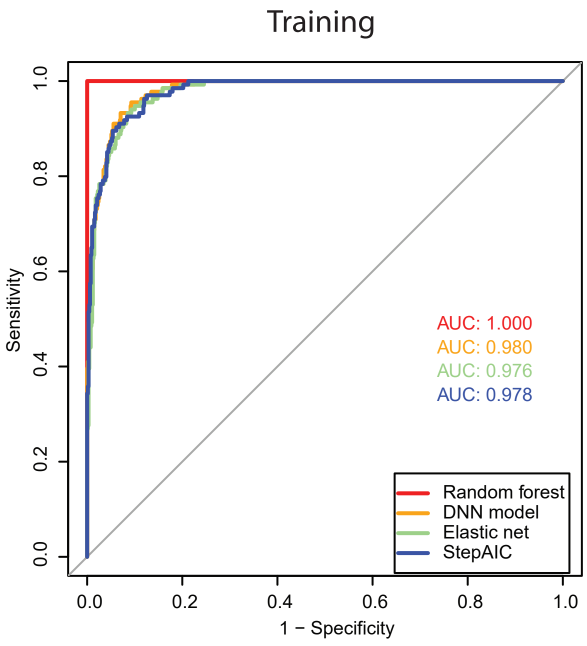

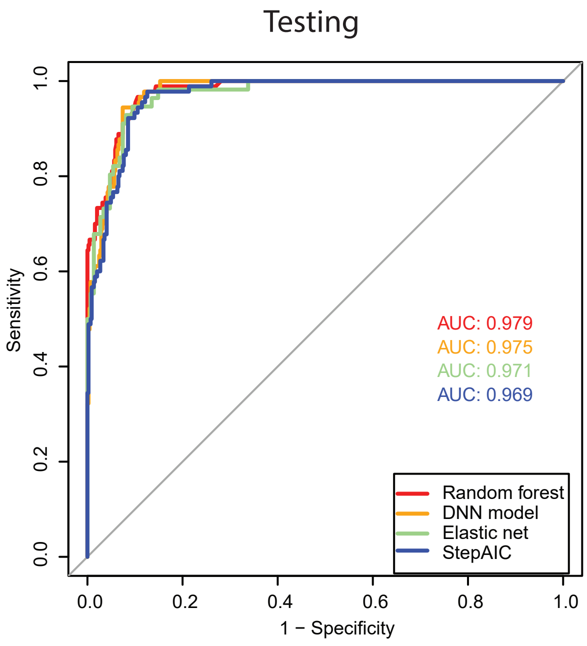

Supplementary Figure 3.

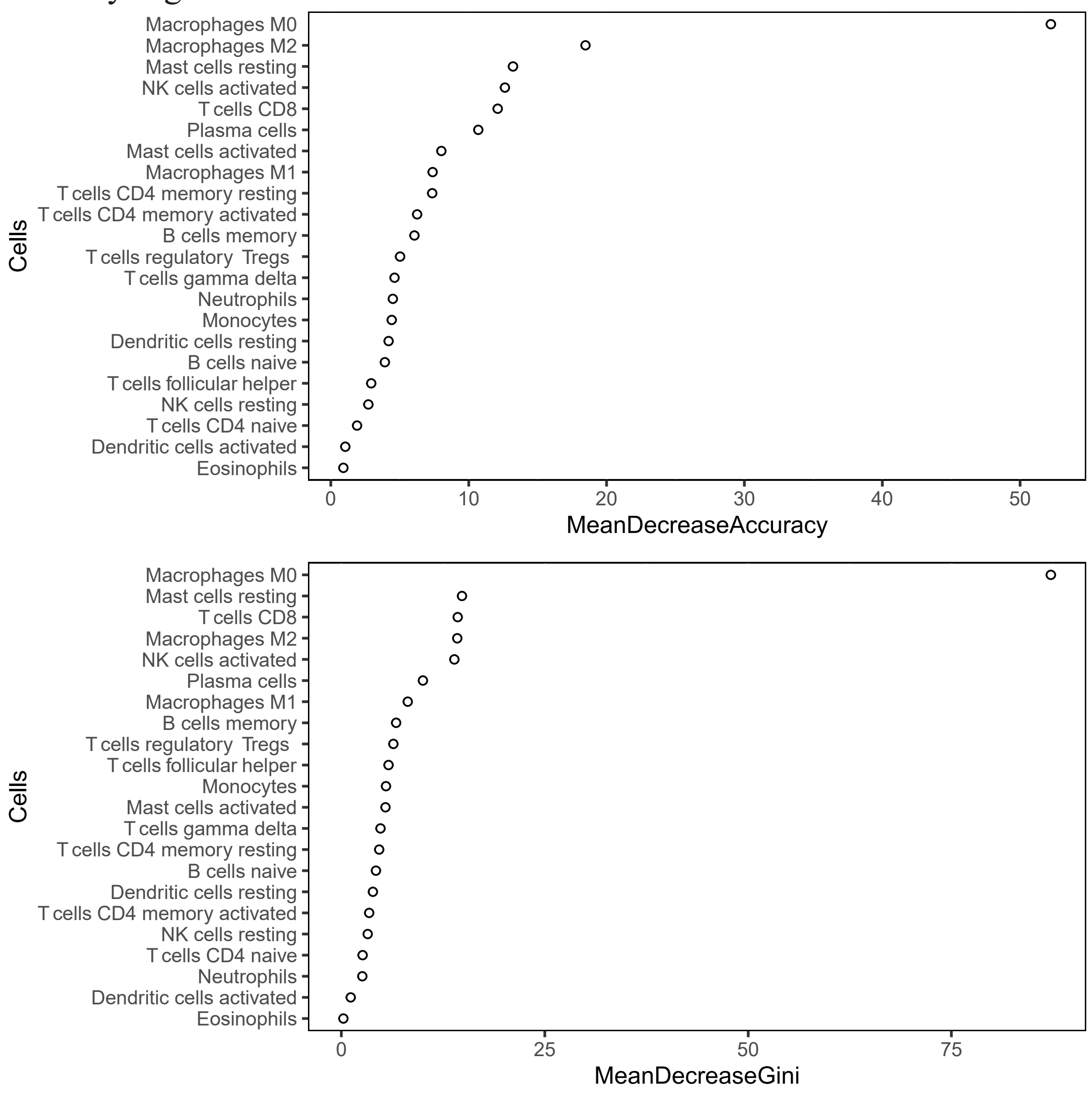

Supplementary Figure 4

A

Metabric

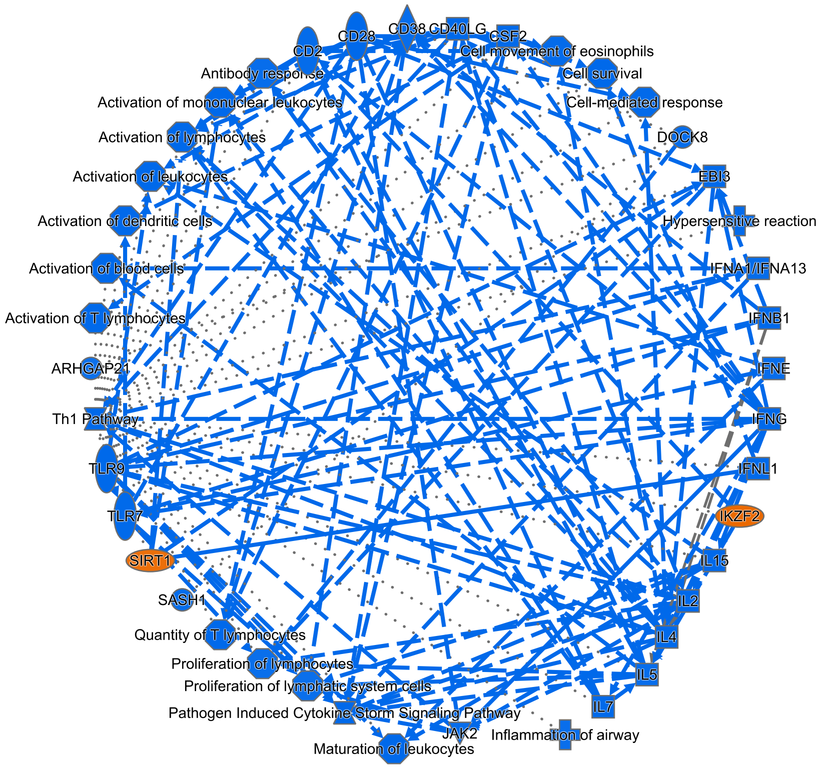

B

TCGA

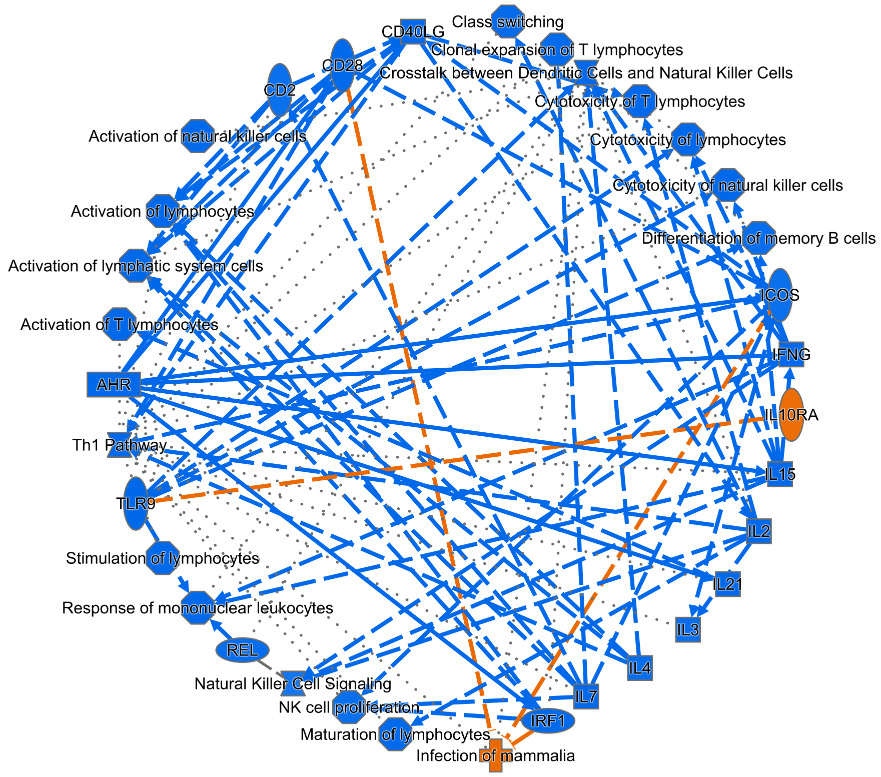

Supplementary Table 1. Elastic net and stepAIC regression models for predicting immune cell subtype clusters in METABRIC

**Elastic net model**			**stepAIC model**	
**Variable**	**coefficient**		**Variable**	**coefficient**
(Intercept)	-5.2176564		(Intercept)	24.845
B cells naive	-1.6929311		B cells naive	-49.827
B cells memory	-1.3572613		B cells memory	-43.861
Plasma cells	-1.8340779		Plasma cells	-37.108
T cells CD8	-2.9320872		T cells CD8	-39.66
T cells CD4 memory resting	4.855054		T cells CD4 naive	-23.216
T cells CD4 memory activated	-7.2425466		T cells CD4 memory resting	-15.199
T cells regulatory Tregs	-1.9584155		T cells CD4 memory activated	-56.705
NK cells activated	-2.0833505		T cells follicular helper	-29.84
Macrophages M0	18.1484351		T cells regulatory Tregs	-41.416
Macrophages M2	-0.4273408		T cells gamma delta	-32.255
Mast cells resting	-2.3535755		NK cells resting	-46.437
Mast cells activated	1.6185543		NK cells activated	-35.993
Neutrophils	-20.4136521		Monocytes	-33.832
			Macrophages M1	-31.136
			Macrophages M2	-38.52
			Dendritic cells resting	-46.944
			Mast cells resting	-36.848
			Mast cells activated	-22.208
			Neutrophils	-127.257

R code:

```
library(factoextra)
library(caret)
library(caTools)
library(randomForest)
library(ROCR)
library(survcomp)
#Import METABRIC  data
METABRIC.ori<read.csv("./metabric_cibersort_100perm.csv", header=TRUE)
METABRIC.sub <- subset(METABRIC.ori, P.value < 0.05)
METABRIC<-METABRIC.sub[,c(2:23)]
#Cluster in METABRIC
METABRIC.dist<- dist(METABRIC, method="euclidean")
METABRIC.clust<-hclust(METABRIC.dist, method = "complete")
plot(METABRIC.clust,hang = -1)
out.METABRIC.clust.id<- cutree(METABRIC.clust, k=2)
table(out.METABRIC.clust.id)
METABRIC.sub$hcluster<-out.METABRIC.clust.id
table(METABRIC.sub$hcluster)
#Split METABRIC  into training and testing datasets
set.seed(123456)
sample = sample.split(METABRIC$hcluster, SplitRatio = 0.6)
train = subset(METABRIC, sample == TRUE)
test  = subset(METABRIC, sample == FALSE)
#Build Random Forest model in training data
train$hcluster<- as.factor(train$hcluster)
set.seed(2)
rf<-randomForest(hcluster~., data=train, importance=TRUE, proximity= TRUE)
importance(rf)
```

```
plot(rf)
par(family="serif", cex=1.1,font=2, cex.lab=1.1, font.lab=1, cex.axis=1.1, font.axis=1 )
varImpPlot(rf)
#Draw ROC for training data
train.predict <- predict(rf, train[-23], type="prob")
pred_train_rf = prediction(train.predict[,2],train$hcluster)
perf_train = performance(pred_train_rf,"tpr","fpr")
plot(perf_train, col='blue',lty=2)
auc <- performance(pred_train_rf,'auc')
auc = unlist(slot(auc,"y.values"))
plot(perf_train,
    xlim=c(0,1), ylim=c(0,1),col='red',
    main=paste("ROC curve (", "AUC = ",auc,")"),
    lwd = 2, cex.main=1.3, cex.lab=1.2, cex.axis=1.2, font=1.2)
abline(0,1)
#Draw ROC for testing  data
test.predict <- predict(rf, test[-23], type="prob")
pred_test_rf = prediction(test.predict[,2],test$hcluster)
perf_test = performance(pred_test_rf,"tpr","fpr")
plot(perf_test, col='blue',lty=2)
auc <- performance(pred_test_rf,'auc')
auc = unlist(slot(auc,"y.values"))
plot(perf_test,
    xlim=c(0,1), ylim=c(0,1),col='red',
    main=paste("ROC curve (", "AUC = ",auc,")"),
    lwd = 2, cex.main=1.3, cex.lab=1.2, cex.axis=1.2, font=1.2)
abline(0,1)
# KM plot for METABRIC  data
OS<-METABRIC.sub$OS
death<-METABRIC.sub$death
```

```
RFS<-METABRIC.sub$RFS

relapse<-METABRIC.sub$Relapse


hcluster<-METABRIC.sub$hcluster

data.os<-data.frame("surv.time"=as.vector(death/12), "surv.event"=as.vector(OS),
                "strat"=as.vector(hcluster))

km.coxph.plot(formula.s=Surv(surv.time, surv.event) ~ strat, data.s=data.os,
        x.label="Time (years)", y.label="OS",
        main.title="", leg.text=paste(c("hcluster_1","hcluster_2"),"", sep=""),
        leg.pos="bottomleft", leg.inset=F, .col=c("blue","red"),leg.bty="n",
        .lty=c(1,1),.lwd=c(3,3),show.n.risk=TRUE, n.risk.step=3, n.risk.cex=0.8, verbose=F)


data.dfs<-data.frame("surv.time"=as.vector(relapse/12), "surv.event"=as.vector(RFS),
                "strat"=as.vector(hcluster))

km.coxph.plot(formula.s=Surv(surv.time, surv.event) ~ strat, data=data.dfs,
        x.label="Time (years)", y.label="DFS",
        main.title="", leg.text=paste(c("hcluster_1","hcluster_2"),"", sep=""),
        leg.pos="bottomleft", leg.inset=F, .col=c("blue","red"),leg.bty="n",
        .lty=c(1,1),.lwd=c(3,3),show.n.risk=TRUE, n.risk.step=3, n.risk.cex=0.8, verbose=F)

# Univariate analysis in TCGA

coxph(Surv(relapse,RFS==1)~hcluster)

coxph(Surv(death,OS==1)~hcluster)

# Multivariate COX regression analysis in METABRIC data

fit_RFS <- coxph(formula = Surv(relapse, RFS == 1) ~ Age + as.factor(ER) + as.factor(PR) +
as.factor(Grade) + as.factor(Stage) + hcluster, data = METABRIC.sub)

summary(fit_RFS)


fit_os <- coxph(formula = Surv(death, OS == 1) ~ Age + as.factor(ER) + as.factor(PR) + as.factor(Grade) +
as.factor(Stage) + hcluster, data = METABRIC.sub)
```

summary(fit_os)

# Apply RF model to TCGA data

```
tcga_predict_class <- predict(rf, tcga_ciber_sub[,c(3:24)], type="class")

tcga_ciber_sub$rf_predi_class <- tcga_predict_class

#KM plot for TCGA data

RFS<-as.numeric(tcga_ciber_sub$DFS)

relapse<-tcga_ciber_sub$Disease_Free

rf_predi_class<-tcga_ciber_sub$rf_predi_class

OS<-as.numeric(tcga_ciber_sub$OS)

death<-tcga_ciber_sub$Overall

data.test<-data.frame("surv.time"=as.vector(relapse/12), "surv.event"=as.vector(RFS),
        "strat"=as.vector(rf_predi_class))

km.coxph.plot(formula.s=Surv(surv.time, surv.event) ~ strat, data.s=data.test,
    x.label="Time (years)", y.label="RFS",
    main.title="", leg.text=paste(c("Cluster 1","Cluster 2"),"", sep=""),
    leg.pos="topright", leg.inset=F, .col=c("blue","red"),leg.bty="n",
    .lty=c(1,1),.lwd=c(3,3),show.n.risk=TRUE, n.risk.step=1, n.risk.cex=1, verbose=F, xlim = c(0,16))


data.test<-data.frame("surv.time"=as.vector(death/12), "surv.event"=as.vector(OS),
        "strat"=as.vector(rf_predi_class))

km.coxph.plot(formula.s=Surv(surv.time, surv.event) ~ strat, data.s=data.test,
    x.label="Time (years)", y.label="OS",
    main.title="", leg.text=paste(c("Cluster 1","Cluster 2"),"", sep=""),
    leg.pos="topright", leg.inset=F, .col=c("blue","red"),leg.bty="n",
    .lty=c(1,1),.lwd=c(3,3),show.n.risk=TRUE, n.risk.step=1, n.risk.cex=1, verbose=F)

# Univariate analysis in TCGA

fit_DFS <- coxph(Surv(Disease_Free,DFS==1) ~  rf_predi_class, data = tcga_ciber_sub)

summary(fit_DFS)

fit_os <- survfit(Surv(Overall, OS) ~ predi_class, data, data = tcga_ciber_sub)
```

```
summary(fit_os)


# Multivariate COX regression analysis in TCGA
tcga_ciber_sub$ER <- as.factor(tcga_ciber_sub$ER)
tcga_ciber_sub$PR <- as.factor(tcga_ciber_sub$PR)
tcga_ciber_sub$Stage <- as.factor(tcga_ciber_sub$Stage)
tcga_ciber_sub$Histology <- as.factor(tcga_ciber_sub$Histology)
tcga_ciber_sub$rf_predi_class <- as.factor(tcga_ciber_sub$rf_predi_class)
fit_DFS <- coxph(Surv(Disease_Free,DFS==1) ~  Age + ER +PR + Stage + Histology + rf_predi_class, data =
tcga_ciber_sub)
summary(fit_DFS)
fit_os <- coxph(Surv(Overall,OS==1) ~  Age + ER +PR + Stage + Histology + rf_predi_class, data =
tcga_ciber_sub)
summary(fit_os)
```

# Apply RF model to Turin data

```
new_bc.predict_class <- predict(rf, new_bc[,c(1:22)], type="class")
new_bc_k_cluster$rf_predi_class <- new_bc.predict_class
#KM plot in Turin data
RFS<-as.numeric(new_bc_k_cluster$Relapse)
relapse<-new_bc_k_cluster$DFS
OS<-as.numeric(new_bc_k_cluster$Death)
death<-new_bc_k_cluster$OS
rf_predi_class<-new_bc_k_cluster$rf_predi_class
data.dfs<-data.frame("surv.time"=as.vector(relapse/12), "surv.event"=as.vector(RFS),
                "strat"=as.vector(rf_predi_class))
km.coxph.plot(formula.s=Surv(surv.time, surv.event) ~ strat, data=data.dfs,
        x.label="Time (years)", y.label="DFS",
        main.title="", leg.text=paste(c("RF_predi_class_1","RF_predi_class_2"),"", sep=""),
        leg.pos="bottomleft", leg.inset=F, .col=c("blue","red"),leg.bty="n",
        .lty=c(1,1),.lwd=c(3,3),show.n.risk=TRUE, n.risk.step=3, n.risk.cex=0.8, verbose=F)
```

```
data.os<-data.frame("surv.time"=as.vector(death/12), "surv.event"=as.vector(OS),
                    "strat"=as.vector(rf_predi_class))

km.coxph.plot(formula.s=Surv(surv.time, surv.event) ~ strat, data.s=data.os,
            x.label="Time (years)", y.label="OS",
            main.title="", leg.text=paste(c("RF_predi_class_1","RF_predi_class_2"),"", sep=""),
            leg.pos="bottomleft", leg.inset=F, .col=c("blue","red"),leg.bty="n",
            .lty=c(1,1),.lwd=c(3,3),show.n.risk=TRUE, n.risk.step=3, n.risk.cex=0.8, verbose=F)
# Univariate analysis in Turin data
RFS<-as.numeric(new_bc_k_cluster$Relapse)
relapse<-new_bc_k_cluster$DFS
OS<-as.numeric(new_bc_k_cluster$Death)
death<-new_bc_k_cluster$OS
coxph(Surv(DFS,Relapse==1) ~rf_predi_class, data = new_bc_k_cluster)
coxph(Surv(OS,Death==1) ~ rf_predi_class, data = new_bc_k_cluster)
# Multivariate COX regression analysis in TCGA
new_bc_k_cluster$E2grp <- as.factor(new_bc_k_cluster$E2grp)
new_bc_k_cluster$PGgrp <- as.factor(new_bc_k_cluster$PGgrp)
new_bc_k_cluster$stage_3_grp <- as.factor(new_bc_k_cluster$stage_3_grp)
new_bc_k_cluster$GRADE <- as.factor(new_bc_k_cluster$GRADE)
new_bc_k_cluster$nhist <- as.factor(new_bc_k_cluster$nhist)
new_bc_k_cluster$rf_predi_class <- as.factor(new_bc_k_cluster$rf_predi_class)
fit_relapse <- coxph(Surv(DFS,Relapse==1) ~ AgeNew + E2grp + PGgrp + stage_3_grp + GRADE + nhist +
rf_predi_class, data = new_bc_k_cluster)
summary(fit_relapse)
fit_death <- coxph(Surv(OS,Death==1) ~ AgeNew + E2grp + PGgrp + stage_3_grp + GRADE + nhist +
hcluster, data = new_bc_k_cluster)
```

```
summary(fit_death)
```
